# Is Three Company or a Crowd? Comparing and Contrasting U.S. and European *Clostridioides* *difficile* Clinical Practice Guidelines

**DOI:** 10.3390/antibiotics11091247

**Published:** 2022-09-14

**Authors:** Jordan Jones, Aditya Pradhan, Morgan E. Pizzuti, Christopher M. Bland, P. Brandon Bookstaver

**Affiliations:** 1 College of Pharmacy, University of South Carolina, Columbia, SC 29208, USA; 2 Prisma Health Richland, Columbia, SC 29203, USA; 3 College of Pharmacy, University of Georgia, Savannah, GA 30602, USA

**Keywords:** *Clostridioides difficile*, guidelines, ACG, ESCMID, IDSA, fidaxomicin, vancomycin, bezlotoxumab, FMT

## Abstract

In 2021, the American College of Gastroenterology (ACG), the Infectious Diseases Society of America in conjunction with the Society for Healthcare Epidemiology of America (IDSA/SHEA), and the European Society of Clinical Microbiology and Infectious Diseases (ESCMID) published updated clinical practice guidelines (CPGs) for the management of *Clostridioides difficile* infections. The differences, sometimes subtle, between these guideline recommendations have caused some debate among clinicians. This paper delves into select key recommendations from each respective CPG and analyzes the differences and evidence associated with each. One primary difference between the CPGs is the preference given to fidaxomicin over vancomycin for initial treatment in non-severe and severe disease endorsed by IDSA/SHEA and ESCMID guidelines, while the ACG-sponsored CPGs do not offer a preference. The emphasis on cost effective data was also a noticeable difference between the CPGs and thus interpretation of the available evidence. When using guidelines to help support local practice or institutional treatment pathways, clinicians should carefully balance CPG recommendations with local patient populations and feasibility of implementation, especially when multiple guidelines for the same disease state exist.

## 1. Introduction

*Clostridioides difficile* infections (CDIs) pose a burden on healthcare systems with significant morbidity, mortality, and healthcare-associated costs. In the United States in 2011, CDIs led to nearly 29,000 deaths, with an estimated incidence of 453,000 cases [1], and it is named an urgent threat by the Centers for Disease Control and Prevention [2]. Outcomes associated with CDI appear to be improving over time. In a study conducted by Shrestha and colleagues, authors found that CDI hospital-related discharges increased from 2004 to 2014, while associated death decreased from 3.6% to 1.6% during the same time period [3]. The increasing incidence of CDIs also leads to an increase in healthcare system costs associated with the management and treatment of these patients. A study conducted by Mollard and colleagues in the US among hospital discharge records from 2012 through 2016 found that hospitalization due to CDI led to an average cost of $10,528, with an average length of stay of six days [4]. Recurrent CDI brings its own challenges, as up to 20% of patients with an initial CDI experience a recurrence. Some patients experience multiple recurrences, unfortunately spiraling into a state of chronic symptoms and antibiotic dependence [5]. These findings demonstrate a need for targeted, guideline-driven quality management of patients with CDI. Although the incidence and burden of healthcare-associated CDI has been steadily decreasing since 2011, clinicians need to remain current on prevention and management strategies [6].

Multiple clinical practice guidelines (CPGs) for the management of CDI were updated in 2021, including guidelines from the American College of Gastroenterology (ACG), the Infectious Diseases Society of America (IDSA) in collaboration with the Society for Healthcare Epidemiology of America (SHEA), and the European Society of Clinical Microbiology and Infectious Diseases (ESCMID). These CPGs were previously published in 2013 [7], 2017 [8], and 2014 [9], respectively. Clinicians highly anticipated updates to each of these CPGs, which were all published in 2021 after the U.S. Food and Drug Administration (FDA) and European Medicines Agency (EMA) approval of bezlotoxumab (BEZ) and additional evidence became available for other treatment modalities. While represented societies and CPG panels are highly respected, discrepancies among their recommendations have caused debate and significant discussion among clinicians. This paper serves to compare and contrast differing select recommendations among the ACG, IDSA/SHEA, and ESCMID CPGs to allow for highly informed decisions in CDI management. We reviewed each of the 2021 CPGs, their previous editions, and their references to determine significant similarities as well as differences among the recommendations and what may have led to the discrepancies.

## 2. Guideline Highlights

The 2017 IDSA/SHEA CPGs were a comprehensive document that included recommendations for the management and prevention of CDIs in both pediatric and adult patient populations. Likewise, the 2013 ACG and ESCMID CPGs also addressed all aspects of care for CDIs. The 2017 IDSA/SHEA, 2013 ACG, and 2014 ESCMID CPGs were concordant in most of their major recommendations, with the primary exception being the use of metronidazole to treat mild or moderate CDI, which was recommended only in the ACG and ESCMID CPGs. The recent updates for each of the CPGs are similar; however, the 2021 IDSA/SHEA CPGs recommend fidaxomicin as the preferred agent throughout, the 2021 ACG CPGs list both vancomycin and fidaxomicin as acceptable first line options, and the 2021 ESCMID CPGs list fidaxomicin as the preferred agent in the initial occurrence but differ from both ACG and IDSA/SHEA in its recommendations for the management of recurrent CDI.

The 2021 ACG CPGs are a comprehensive set of recommendations that address both primary and secondary prevention, diagnosis, treatment, and special populations. The 2021 IDSA/SHEA CPGs are a focused update; therefore, the 2017 CPGs still house the majority of their recommendations, with the exception of the three major changes: the use of vancomycin versus fidaxomicin during an initial CDI occurrence, the use of vancomycin versus fidaxomicin during recurrent CDIs, and the role of BEZ in conjunction with standard of care antibiotics. The 2021 ESCMID CPGs are an update of the 2014 CPGs and aim to answer nine specific CDI-related questions, which encompasses the majority of CDI management issues.

The recommendations included in each of the CPGs were assigned strength and quality measures according to the Grading of Recommendations Assessment, Development, and Evaluation (GRADE) [10] approach by a GRADE methodologist, with the IDSA/SHEA and ESCMID authors using the GRADEpro guideline development software [11] to aid in decision-making. The ACG CPGs explain that a recommendation is considered “strong” when the benefit outweighs the risk and is considered “conditional” when there is uncertainty about the relationship between benefit and risk [12]. The IDSA/SHEA CPGs use the phrase, “we recommend” to provide strong recommendations, which they categorize as one that most individuals should receive; they use, “we suggest” to provide conditional recommendations, in which shared clinical decision making should be used during management [13]. The ESCMID CPGs use “strong” and “weak” to characterize the strength of recommendation, and use very low, low, moderate, or high to express the quality of evidence; these CPGs also include a “good practice statement”, which is described as an expert opinion for instances where guidance is necessary but evidence is not sufficient to make an official recommendation [14]. The ACG CPGs categorize the levels of evidence as follows: “high” indicates a decision that will likely not be changed in the presence of new data, “moderate” indicates a decision where new data will likely have an impact, and “low” indicates a decision that is likely to change in the presence of new data [12].

The ACG CPG author panel included seven individuals, some of whom currently provide patient care and are leading scholars in fecal microbiota transplant (FMT), with only one having a formal background in infectious diseases. The IDSA/SHEA CPG author panel also included seven individuals, with the majority having a background in infectious diseases. The ESCMID CPG author panel comprised of 20 individuals across 11 countries in Europe, the majority of which have a background in microbiology and/or infectious diseases, and only one having a background in gastroenterology; however, these authors also incorporated the expertise of external consultants, which included multiple gastroenterologists. Unsurprisingly, only one author of the ACG CPG has an infectious diseases background and only one author of the IDSA/SHEA CPG has a gastroenterology background. The authors of each CPG are represented by both physicians and pharmacists, which provides a value-added expert level review [15,16].

### 2.1. Financial Analysis

There is a stark contrast between how each of the CPGs address the financial components of each therapeutic option, which may have impacted each organization’s recommendations. The 2021 ESCMID CPGs did not make their recommendations based on economic considerations; however, they acknowledged that access may be limited and offered alternative recommendations in the instance where first line therapies are not accessible. The ESCMID CPGs stated that recommendations were based on clinical cure, recurrence, and sustained cure [14].

Research surrounding fidaxomicin included multiple cost-effectiveness models between 2016 and 2020 that were referenced in the ACG and IDSA/SHEA CPGs. Notably, each of these CPGs reference different cost-effectiveness analyses, which may be a factor in the differing recommendations. Previous CPGs from the ACG in 2013 did offer cost comparison among the recommended agents (detailed below). The 2021 ACG CPGs, which referenced four cost-effectiveness analyses, mention that although fidaxomicin is the more expensive option upfront, the associated decrease in 30-day recurrence will offset the initial cost, giving each regimen a similar overall cost-effectiveness [12,17,18]. The 2021 IDSA/SHEA CPGs referenced seven cost-effectiveness analyses and go into much detail about their results, which they use to support the preference for fidaxomicin over vancomycin. The recommendations included in the IDSA/SHEA CPGs use the phrase, “the cost-effectiveness analysis probably favors the use of fidaxomicin”, which contrasts with the phrasing of the 2021 ACG CPG, “leading to near equivalence with vancomycin”, [12,13].

### 2.2. Literature Analysis

The 2021 IDSA/SHEA CPGs offer recommendations for three major components of CDI management: treatment of index CDI, treatment of recurrent CDI, and the use of BEZ to prevent future recurrence. Authors formed PICO questions to address each of these sections, and used six randomized controlled trials (RCTs) to support their recommendations [19,20,21,22,23,24]. Notably, four of their six sources were RCTs, which were either supported by or many authors declared conflicts of interest (COI) with Merck & Co. Inc. or Optimer Pharmaceuticals, whose product line of fidaxomicin and BEZ was later acquired by Merck [19,22,23,24]. The other two RCTs were supported by or had multiple conflicts of interest with Astellas Pharmaceuticals [20,21]. These CPGs also rely heavily on cost-effectiveness analyses, four of the seven cited were industry-funded, with another having authorship with a declared COI with Cubist Pharmaceuticals, which was acquired by Merck & Co., Inc. [25,26,27,28,29,30,31]. Both the 2021 ACG and ESCMID CPGs reference these same RCTs when supporting their recommendations; however, they do not allow previously discussed financial considerations to guide their recommendations.

## 3. Therapeutic Options

Each of the 2021 CPGs address similar clinical questions but do not always agree on their recommendations. The main differences between these CPGs are summarized in Table 1, with an in-depth discussion following in subsequent sections.

### 3.1. Fidaxomicin Versus Vancomycin in Initial Occurrence

Previous 2017 IDSA/SHEA CPGs recommended either vancomycin or fidaxomicin for the management of non-severe CDIs, and each of these agents were favored over metronidazole [8]. This change to the IDSA/SHEA guidance is the most dramatic change that has occurred within these CPGs, with the new recommendation of fidaxomicin as the preferred therapy. However, vancomycin is listed as an acceptable alternative if resources do not allow for the use of fidaxomicin. Metronidazole remains a non-preferred treatment option, only to be used when vancomycin and fidaxomicin are not available or contraindicated. The 2021 IDSA/SHEA recommendation explicitly states fidaxomicin as the preferred antibiotic (conditional, moderate certainty), which is guided by the lower rate of recurrence at 30 days compared to vancomycin, although both agents demonstrate comparable initial clinical cure rates [13]. Of further note, fidaxomicin has a narrower spectrum of activity for enteric commensals and is potentially more cost-effective than vancomycin due to the decreased recurrence at 30 days [25,26]. It is speculated that fidaxomicin use will have higher compliance rates compared to vancomycin due to administration frequency. Although not explicitly discussed in either CPG, data continues to emerge on elevated vancomycin MICs in *C. difficile* isolates, which may influence future application of these guideline recommendations if clinical outcome correlations are verified [32].

Previous ACG CPGs published in 2013 recommended metronidazole to treat mild to moderate CDIs, while vancomycin was reserved for severe infections. Those CPGs mentioned the newly FDA approved fidaxomicin; however, withheld it from formal recommendations due to lack of evidence and cost. The 2013 CPGs compared the cost of each treatment, pricing for a full ten-day course was as follows: metronidazole $22, vancomycin capsules $680, vancomycin oral solution compounded from intravenous (IV) solution components ranged $100–400, and fidaxomicin $2800 [7]. The 2021 ACG CPGs list both vancomycin (strong recommendation, low quality of evidence) and fidaxomicin (strong recommendation, moderate quality of evidence) as first line options to treat non-severe CDI, and although the CPGs do not explicitly state that vancomycin is preferred over fidaxomicin, the recommendations are listed in such order that vancomycin is listed first, which may influence interpretation of value placed on the recommendation itself. The ACG CPGs also list metronidazole as an alternative to vancomycin and fidaxomicin in non-severe low-risk patients (strong recommendation, moderate quality of evidence) [12].

Previous 2014 ESCMID CPGs recommended the use of metronidazole for the treatment of an initial non-severe CDI episode, with vancomycin and fidaxomicin being equally reserved for the treatment of severe infections. This recommendation was led by the absence of statistical significance between metronidazole and vancomycin [9]. The 2021 CPGs recommend fidaxomicin as the preferred treatment for initial non-severe CDI (strong recommendation, moderate certainty of evidence). If fidaxomicin is not available for use, ESCMID recommends vancomycin as an alternative (strong recommendation, high certainty of evidence), followed by metronidazole if vancomycin is not available (strong recommendation, moderate certainty of evidence).

The preference for fidaxomicin is guided by a decrease in recurrence. The ESCMID CPG differs from other CPGs by including a recommendation for the use of BEZ during the initial episode if patients are unable to receive fidaxomicin as the standard of care antibiotic and are at high risk for recurrence. The authors exclude fidaxomicin from the BEZ combination because of lack of data; only 4% of patients in the MODIFY trials received fidaxomicin/BEZ, compared to the 48% which received vancomycin/BEZ [14,22]. In severe CDI, the ESCMID CPGs note a lack of data supporting superiority; therefore, both vancomycin and fidaxomicin are equally preferred (good practice statement) [14].

### 3.2. Recurrence

Because a previous episode of CDI is a well-established risk factor for recurrence, the CPG recommendations are separated by the number of recurrences the patient has experienced.

#### 3.2.1. Initial Recurrence

For the initial recurrence episode, the 2017 IDSA/SHEA CPG recommendations were dependent upon the therapy used to manage the initial infection. If metronidazole was used, IDSA/SHEA CPGs recommended treatment with vancomycin. However, if vancomycin was used, prolonged taper and pulse dosed vancomycin or fidaxomicin was recommended [8]. The 2021 IDSA/SHEA CPGs updated this recommendation to fidaxomicin as the preferred treatment for initial recurrence (conditional recommendation, moderate quality of evidence), irrespective of initial treatment agent with exception of metronidazole where vancomycin standard therapy is provided as an option for consideration. However, vancomycin taper, pulse, or standard regimens are acceptable alternatives, regardless of the therapy used during the index infection. The 2021 IDSA/SHEA CPGs highlight studies using a tapered and pulse regimen fidaxomicin [33,34] but do not include this regimen as a formal recommendation due to lack of comparative studies.

The 2013 ACG CPGs recommended repeating the same regimen for the first recurrence that was used during the initial infection, unless recurrence was severe, then vancomycin was preferred. The 2021 ACG CPGs update this recommendation to prefer vancomycin regardless of the agent used during the initial infection (strong recommendation, very low quality of evidence), or fidaxomicin as long as it was not used previously (strong recommendation, moderate quality of evidence).

The 2014 ESCMID CPGs recommended vancomycin and fidaxomicin as equally preferred agents for the first recurrence of CDI. The 2021 CPGs offer recommendations based on the previous regimen. If vancomycin was used to treat the initial CDI, then a standard fidaxomicin regimen is recommended for the first recurrence (strong recommendation, low certainty of evidence). If fidaxomicin was used to treat the initial CDI, then BEZ should be added to either vancomycin (weak recommendation, moderate certainty of evidence) or fidaxomicin (good practice statement). If neither BEZ nor fidaxomicin are available to the patient, a vancomycin taper and pulse regimen may be used (weak recommendation, very low certainty of evidence).

#### 3.2.2. Subsequent Recurrences

For patients experiencing multiple recurrences, the 2017 IDSA/SHEA CPGs recommended tapered and pulsed vancomycin, vancomycin followed by rifaximin, or fidaxomicin as antibiotic therapies with no preference given among the options. If patients continued to experience CDI recurrence despite appropriate antibiotic regimens, these CPGs recommended FMT as the next step [8]. The 2021 IDSA/SHEA CPGs now recommend fidaxomicin as the preferred treatment for multiple recurrences, regardless of the agent used during previous infections (conditional recommendation, low quality of evidence).

The 2013 ACG CPGs recommended management of the second recurrence with pulsed vancomycin, then an FMT beginning with the third recurrence [7]. The 2021 ACG CPGs also recommend consideration of FMT (strong recommendation, moderate quality of evidence) for the second recurrence. If the patient is not a candidate for FMT, the ACG CPGs recommend the use of once daily suppressive vancomycin to prevent future CDI recurrence (conditional recommendation, very low quality of evidence), acknowledging that some patients may require twice or three times daily dosing if they continue to experience loose stools; this recommendation is drawn from a small retrospective study [35]. As with the treatment recommendations for the initial occurrence, ACG does not state a preference between vancomycin and fidaxomicin; however, vancomycin is once again listed before fidaxomicin [13].

The 2014 ESCMID CPGs recommend tapered and pulsed regimen of vancomycin and standard regimen of fidaxomicin equally for the treatment of recurrent CDI, or an FMT following four days of vancomycin therapy [9]. The 2021 CPG recommendations are dependent on the regimen used during the first recurrence. If fidaxomicin was used alone, then BEZ is recommended in conjunction with either vancomycin or fidaxomicin (weak recommendation, low certainty of evidence); if a BEZ combination regimen was used during the first recurrence, an FMT should be used (weak recommendation, moderate certainty of evidence) [14].

### 3.3. Role of Bezlotoxumab

None of the prior CPGs mention BEZ as a treatment option as literature review was completed prior to the FDA and EMA approvals. BEZ is administered as a single-dose IV infusion and is only indicated to prevent recurrence of CDI, therefore it should only be used in conjunction with standard of care antibiotics [36]. The MODIFY trials showed that, although the combination of BEZ with standard of care antibiotics resulted in a similar clinical cure, there was a 10% reduced risk of recurrence in patients with at least three risk factors [22,23].

The 2021 IDSA/SHEA CPGs encourage the use of BEZ in addition to standard of care antibiotics if the patient experiences a recurrent CDI within six months of the previous infection (conditional recommendation, very low certainty of evidence). However, this recommendation is backed by limited evidence for the use of BEZ combination therapy with fidaxomicin [13].

The 2021 ACG CPGs state that BEZ showed no significant benefit over placebo in low-risk patients and, therefore, should be reserved for the prevention of CDI recurrence in patients who are considered high risk [23]. High risk patients are described as being over the age of 65 with one or more of the following: recurrence within six months, severe CDI, or immunocompromised state (conditional recommendation, moderate quality of evidence) [12].

Each of the 2021 CPGs mention the increased incidence of heart failure in the BEZ arm of the MODIFY trials [23] but differ slightly in their recommendations. The IDSA/SHEA and ESCMID CPGs only recommend BEZ in patients with congestive heart failure (CHF) if the benefits outweigh the risks. The 2021 ACG CPGs do not recommend the use of BEZ in patients with CHF and recommend using caution in patients with severe underlying cardiovascular abnormalities, which the IDSA/SHEA CPGs do not address.

### 3.4. Role of Fecal Microbiota Transplant

The process of FMT introduces donor fecal material into a recipient’s intestinal tract to change the composition of the gut microbiome and make it healthier. The 2021 ACG CPGs note that FMT can result in reduced rates of sepsis and CDI-related colectomy-associated problems [12]. Patients must be screened prior to the administration of FMT to ensure they are a proper candidate. Screening consists of laboratory testing and a patient interview to identify possible risk factors that could lead to poor outcomes from infection or adverse effects; these risk factors include the use of medications that can alter gut microbiome, history of infection, and pre-existing disorders affecting the gut microbiome [37]. Since June 2019, the FDA has published several safety alerts regarding adverse events and warnings associated with FMT, including bacteremia with subsequent death of a patient who acquired *Escherichia coli* [38,39,40].

The 2017 IDSA/SHEA CPGs recommended FMT after the second and subsequent recurrences of CDI (strong recommendation, moderate quality of evidence) and stated that, although FMT may be used for patients with irritable bowel disease (IBD), there is less benefit when compared to patients without IBD [8]. The 2021 IDSA/SHEA CPG recommends the use of FMT for the second recurrence of CDI, after failure of appropriate antibiotic regimens [13].

The 2013 ACG CPGs stated that FMT may be considered during a third CDI occurrence (second recurrence) after an appropriate regimen of pulsed vancomycin was trialed (conditional recommendation, moderate quality evidence) [7]. Likewise, the 2021 ACG CPGs recommend FMT during a third occurrence of CDI (strong recommendation, moderate quality of evidence) but also extends FMT recommendations to include severe and fulminant CDI (strong recommendation, low quality of evidence), and recurrence within 8 weeks (conditional recommendation, very low quality evidence) [12]. The 2021 ACG CPG recommends the administration of FMT via colonoscopy (strong recommendation, moderate quality of evidence) or oral capsules (strong recommendation, moderate quality of evidence) [12].

The 2014 ESCMID CPGs strongly recommended the use of FMT following a four day course of vancomycin after multiple CDI recurrences [9]. The 2021 CPGs maintain a recommendation for FMT during the second or subsequent recurrences. These authors purposely do not endorse a preferred administration technique but recommend the use of products from non-commercial stool banks that regularly follow both the donors and recipients [14].

### 3.5. Role of Probiotics

Probiotic products are marketed as supplements to promote healthy gut microbiota. *Saccharomyces boulardii* demonstrated the potential to be effective in decreasing the recurrence of CDI when supplemented alongside metronidazole or vancomycin; however, there was not enough evidence showing clinical benefit in decreasing recurrence alone [41]. 

The 2017 IDSA/SHEA CPGs stated that there was insufficient evidence to make a formal recommendation regarding probiotics as primary prevention of CDI [8]. The 2021 IDSA/SHEA focused update does not mention the role of probiotics in CDI treatment, so IDSA/SHEA does not make any formal recommendations at this point.

The 2013 ACG CPGs mention limited evidence for probiotics and their role in decreasing the recurrence of CDIs [7]; however, *Lactobacillus rhamnoses GG* and *S. boulardii* may reduce the occurrence of antibiotic-associated diarrheal symptoms. The 2021 ACG CPGs recommend against the use of probiotics for the prevention of CDI in patients, regardless of antibiotic choice or use [12].

The 2014 ESCMID CPGs mentioned that there was not enough evidence to support the use of probiotics to prevent CDI, and the 2021 CPGs now recommend against their use [9,14].

## 4. Conclusions

The ACG, IDSA/SHEA, and ESCMID have all published updated CPGs for the management of CDIs in 2021. The 2021 CPGs have sparked some debate among clinicians because they do not offer the same recommendations. There are many factors that will influence the management of CDIs, a major one being access to resources. For inpatients transitioning at discharge, outpatient pharmacy “meds to bed” services can play a vital role in ensuring the patient receives the appropriate CDI therapy before being discharged from the hospital [42]. Additionally, antimicrobial stewardship programs in the inpatient and outpatient setting can assist with ensuring continuity of care and anticipating challenges due to cost and access, including prior authorization requirements, which certainly influence subsequent adherence [43].

Clinicians should evaluate CPGs, including the evidence used to arrive at the panel recommendations, in the setting of their institution and patient population. Each hospital should certainly have protocols established for CDI management that include inpatient, outpatient, and transitions of care settings. Leveraging interdisciplinary teams is ideal to determine the most appropriate protocols for individual institutions and recommendations for patients who fall out of typical guideline pathways.

## Figures and Tables

**Table 1:** Summary of Differences Among 2021 Clinical Practice Guidelines.

	2021 ACG	Strength/Quality	2021 IDSA/SHEA	Strength/Quality	2021 ESCMID	Strength/Quality
**Initial Occurrence** **(non-severe)**	Vancomycin preferred	Strong/Low	Fidaxomicin preferred	Conditional/Moderate	Fidaxomicin preferred	Strong/Moderate
	Fidaxomicin preferred	Strong/Moderate	Vancomycin alternative	Strong/High ^¥^	Vancomycin alternative	Strong/High
	Metronidazole alternative	Strong/Moderate	Metronidazole alternative	Weak/High ^¥^	Metronidazole alternative	Strong/High
**Initial Occurrence** **(severe)**	Vancomycin preferred	Strong/Low	Fidaxomicin preferred	Conditional/Moderate	Vancomycin or Fidaxomicin equal	Good Practice Statement
	Fidaxomicin preferred	Conditional/Very Low	Vancomycin alternative	Strong/High ^¥^		
**First Recurrence**	Vancomycin preferred	Strong/Very Low	Fidaxomicin preferred	Conditional/Moderate	Fidaxomicin preferred *	Strong/Low
	Fidaxomicin preferred *	Conditional/Moderate	Vancomycin alternative	Weak/Low ^¥^	Vancomycin + BEZ Fidaxomicin + BEZ	Weak/Moderate Good Practice Statement
**Subsequent** **Recurrence(s)**	FMT preferred **	Strong/Moderate	Fidaxomicin preferred	Conditional/Low	FMT preferred	Weak/Moderate
			Vancomycin alternative	Weak/Low ^¥^	Vancomycin + BEZ Fidaxomicin + BEZ	Weak/Low
**Role of BEZ**	High risk only	Conditional/Moderate	Recurrence within 6 months	Conditional/Very Low	High risk initial episode with vancomycin	Weak/Moderate
					Recurrences	Weak/Low
**Role of Fecal Microbiota Transplant (FMT)**	3+ total CDI	Strong/Moderate	3+ total CDI	Strong/Moderate ^¥^	3+ total CDI	Weak/Moderate
**Role of Probiotics**	Recommend against	Conditional/Moderate	No recommendation	-	Recommend Against	Strong/Low

* If not previously used in the treatment of the initial infection. ** Suppressive vancomycin therapy recommended in patient if not a candidate (Conditional/Very Low). ^¥^ Strength/Quality language from 2017 CPGs differs from 2021 focused update.
